# Sensitive Dentine

**Published:** 1897-05

**Authors:** E. T. Darby

**Affiliations:** Philadelphia.


ARTICLE VII.

### SENSITIVE DENTINE.

BY DR. E. T. DARBY, PHILADELPHIA.

I think sensitive dentine a normal condition. All real tissues with rich nervous supply are sensitive. The fibrille undoubtedly have an intimate relationship with the pulp, and any injury to the fibrils is conveyed to the pulp. Healthy dentine is sensitive, but not to the same extent as diseased dentine (decayed dentine).

I do not believe in the theory of inflammation in the dentine. We can not have inflammation without blood-vessels, and there are no blood-vessels in the dentine. The fibrils in the dentine are nerve fibrils, and are a prolongation of the odontoblastic cells on the surface of the pulp.

Hypersensitiveness of the dentine is dependent on the amount of acid present in the decayed structure. This hypersensitiveness may be diminished almost instantly by putting in the cavity of decay a little bicarbonate of soda before beginning to excavate.

Cavities along the labial and buccal surfaces are often more sensitive because they are bathed in an acid mucus from the gingive. It is always well to apply a little bicarbonate of soda before beginning to cut these, especially if the rubber-dam be not first applied.

I don't think the nearness or distance from the pulp has anything to do with the excessive sensibility.

My treatment for sensitive dentine is the following: First, apply rubber-dam. Then pack into cavity for a moment bicarbonate of soda. Then wipe the cavity with absolute alcohol, dry thoroughly with warm air (not hot air), then put into the cavity for a few moments Robinson's (equal parts carbolic acid and caustic potash) and dry the cavity again, and go ahead with sharp excavators. When I have done this I have very little complaint from patients. I don't think cutting from the pulp instead of toward it has anything to do with the promotion or abuse of pain in excavating. Somebody made such an assertion years ago, and it has been handed along from one practitioner to another without question. There is no physiological reason why it should be true, and my experience and observation convinces me that there is nothing in it but theory.

If the dentist will take the time in each case to give preliminary treatment indicted, he will not be troubled much in his effort to excavate sensitive dentine. He may be obliged to renew the application if the decay be deep seated, but it is well worth the time spent.

Dr. Cheney: Apply rubber dam, pack the cavity with bicarbonate of soda for a minute or two, wash out with alcohol, and evaporate with warm air, not hot, using the warm air till dentine is dry. If this does not put the dentine in condition to be worked with sharp instruments without much pain, I place in cavity a pellet of cotton dipped in Dr. Black's, 1, 2. 3 mixture, or some of the essential oils, tannin and glycerin paste, cocain and glycerine paste, or Robinson's remedy, drying out with warm air. This treatment in a majority of cases works very nicely. In mouths known to be strongly acid, or where there are labial or buccal cavities I have the patient use Philip's milk of magnesia as a wash for a few days for the operation. With extremely nervous patients bromid of potassium in ten or fifteen grain doses, three times a day for three or four days, will in many cases work like a charm,

I prefer bromid of potassium to morphin and the coal tar derivatives, though great benefit comes from these many times.

Dr. Craven has well said "Much may be accomplished by discreet impressions made on the mind of the patient in a conversational way." Keep the patient interested in something foreign to the operation.—*Dental Review.*

